# Cellular zinc status alters chromatin accessibility and binding of p53 to DNA

**DOI:** 10.26508/lsa.202402638

**Published:** 2024-07-08

**Authors:** Daniel Ocampo, Leah J Damon, Lynn Sanford, Samuel E Holtzen, Taylor Jones, Mary A Allen, Robin D Dowell, Amy E Palmer

**Affiliations:** 1 Department of Biochemistry, University of Colorado, Boulder, CO, USA; 2 Department of Molecular, Cellular, Developmental Biology, University of Colorado, Boulder, CO, USA; 3 BioFrontiers Institute, University of Colorado, Boulder, CO, USA

## Abstract

Zn^2+^ is an essential metal required by approximately 850 human transcription factors. How these proteins acquire their essential Zn^2+^ cofactor and whether they are sensitive to changes in the labile Zn^2+^ pool in cells remain open questions. Using ATAC-seq to profile regions of accessible chromatin coupled with transcription factor enrichment analysis, we examined how increases and decreases in the labile zinc pool affect chromatin accessibility and transcription factor enrichment. We found 685 transcription factor motifs were differentially enriched, corresponding to 507 unique transcription factors. The pattern of perturbation and the types of transcription factors were notably different at promoters versus intergenic regions, with zinc-finger transcription factors strongly enriched in intergenic regions in elevated Zn^2+^. To test whether ATAC-seq and transcription factor enrichment analysis predictions correlate with changes in transcription factor binding, we used ChIP-qPCR to profile six p53 binding sites. We found that for five of the six targets, p53 binding correlates with the local accessibility determined by ATAC-seq. These results demonstrate that changes in labile zinc alter chromatin accessibility and transcription factor binding to DNA.

## Abstract (precis)

ATAC-seq and transcription factor enrichment analysis reveal that perturbations in labile Zn^2+^ alter chromatin accessibility and enrichment of transcription factor motifs in regions of open chromatin.

## Introduction

Regulation of gene expression is at the heart of virtually every biological process. How genes are switched on and off at a precise time and in a specific place drives developmental programs, defines the type and state of a cell, regulates cell fate decisions, and underlies basic physiology. The ability of transcription factors (TFs) to regulate gene expression is typically proportional to the accessibility of their binding sites in the genome (Lara-Astiaso et al, 2014; Ampuja et al, 2017; Starks et al, 2019). TFs either bind directly at promoters or in distal regulatory regions called enhancers, both of which are characterized by nucleosome depletion. DNase-seq (Song & Crawford, 2010) and ChIP-seq (Barski et al, 2007; Johnson et al, 2007) have long been used to map regions of open chromatin and TF binding, respectively. TF binding can create a footprint of depletion of DNase-seq signal, although it has been reported that 80% of TFs do not show detectable footprints (Baek et al, 2017). For TFs that do show footprints, DNase-seq has the advantage that it can capture TF-binding information for many TFs simultaneously. Consequently, several computational methods have been created to correlate DNase-seq signal with TF-binding motifs (Gusmao et al, 2016; Vierstra & Stamatoyannopoulos, 2016). Because DNase-seq and ChIP-seq require millions of cells to generate high-quality datasets, an alternative approach for directly probing regions of open chromatin is becoming more widely used; the Assay for Transposase Accessible Chromatin with sequencing (ATAC-seq) (Buenrostro et al, 2013; Corces et al, 2017) requires only 500–50,000 cells. The transposase used in ATAC-seq inserts sequencing adapters into regions of open chromatin, and these regions correlate well with regulatory regions identified via DNase-seq and ChIP-seq (Buenrostro et al, 2013; Minnoye et al, 2021). However, ATAC-seq does not allow for straightforward identification of which TFs may be active within these regions of open chromatin. Computational methods to identify TF “footprints” have been developed (Quach & Furey, 2017; Li et al, 2019; Bentsen et al, 2020), but these historically have not been as robust for ATAC-seq datasets as they are for DNase-seq datasets (Quach & Furey, 2017). Further, as previously noted, not all TFs leave detectable footprints, and footprint depth is likely proportional to residence time on DNA (Sung et al, 2016; Baek et al, 2017).

Transcription factor enrichment analysis (TFEA) is a recently developed computational approach that seeks to identify differentially active TFs in genomic datasets (Rubin et al, 2021). TFEA takes regions of active transcription as input and subsequently uses DESeq2 (Love et al, 2014) to establish a ranked list of differentially transcribed regions. Each of these regions is then subjected to TF motif scanning to determine which TF motifs are enriched in regions of open chromatin in a particular treatment, and an enrichment score (E-score) is calculated for each treatment relative to the control. TFEA was applied to nascent transcription datasets and accurately identified transcriptional responses to Nutlin-3a, lipopolysaccharide, and dexamethasone (Rubin et al, 2021). TFEA can also be applied to ATAC-seq datasets to identify TF motifs associated with open chromatin and whether there is an increase or decrease in association in response to a given perturbation (Gupta et al, 2021; Walker et al, 2021). Because regions of active transcription are associated with regions of open chromatin (both at enhancers and promoters), TFEA can thus be used to infer TFs that are enriched or depleted in genomic regions capable of active transcription in response to a given perturbation.

Of the more than 1,600 known human TFs, approximately half (48%) bind zinc (Lambert et al, 2018). Whereas the specific mode of zinc ion (Zn^2+^) coordination can vary, many of these TFs use zinc fingers to bind to DNA. Loss of Zn^2+^ results in a dysfunctional protein, with one of the most well annotated examples being p53 and its tumorigenic R175H mutation (Butler & Loh, 2003; Yu et al, 2014; Chiang et al, 2021). Whereas this is evidence that some TFs may be susceptible to altered activities because of Zn^2+^ levels, historically the metal regulatory transcription factor 1 (MTF1) is the only known TF whose activity is directly titratable by Zn^2+^ within the cell (Westin & Schaffner, 1988; Bittel et al, 1998; Guerrerio & Berg, 2004; Lindert et al, 2009). However, we and others have shown that perturbation of cellular Zn^2+^ across multiple cell types results in global changes in transcription that are not directly related to MTF1 (Hardyman et al, 2016; Sanford et al, 2019; Han et al, 2022). Although the concentrations of Zn^2+^ used in these studies were higher than expected physiological levels, even mild exposure to Zn^2+^ can alter the transcriptional landscape (Sanford et al, 2019). Furthermore, it has been established that the labile Zn^2+^ pool of mammalian cells is dynamic, such that neuronal stimulation (Sanford & Palmer, 2020), cell-cycle progression (Lo et al, 2020; Rakshit et al, 2023), and fertilization (Kim et al, 2011; Que et al, 2015), among other processes, can induce fluctuations to this labile pool in the pM to nM range. However, it is still unknown whether Zn^2+^ modulates transcription directly through its binding to transcription factors or indirectly through signaling pathways such as the MAPK pathway (Anson et al, 2021).

In this study, we used ATAC-seq and TFEA to identify TFs that are putatively activated and/or repressed as a consequence of cellular Zn^2+^ status. Elevation of Zn^2+^ using ZnCl_2_ and depletion of Zn^2+^ using a Zn^2+^-specific chelator caused broad changes in the accessible chromatin landscape, even after a short 30 min perturbation. TFEA revealed 648 motifs, corresponding to 507 unique TFs that were differentially associated with newly open chromatin upon perturbation of Zn^2+^. We found that elevation of Zn^2+^ led to enrichment of TF motifs in regions of open chromatin in distal regulatory regions, and 88% of the TFs were zinc-finger TFs. Conversely, depletion of Zn^2+^ led to strong enrichment of TFs in regions of open chromatin at promoters. To probe how TFEA enrichments correlate with TF occupancy, we selected a candidate TF (p53) and performed ChIP-qPCR. We found that for five of six selected p53 target sites, p53 binding correlated with local accessibility, such that a decrease in accessibility led to decreased p53 binding and an increase in accessibility correlated with increased p53 binding. These results reveal that zinc status alters chromatin accessibility, and this can be further propagated to zinc-dependent changes in TF binding to target genes, indicating that the functionality of zinc-dependent TFs can be sensitive to the labile Zn^2+^ pool.

## Results

### Perturbation of the labile Zn^2+^ pool leads to changes in chromatin accessibility

**Figure 1.**
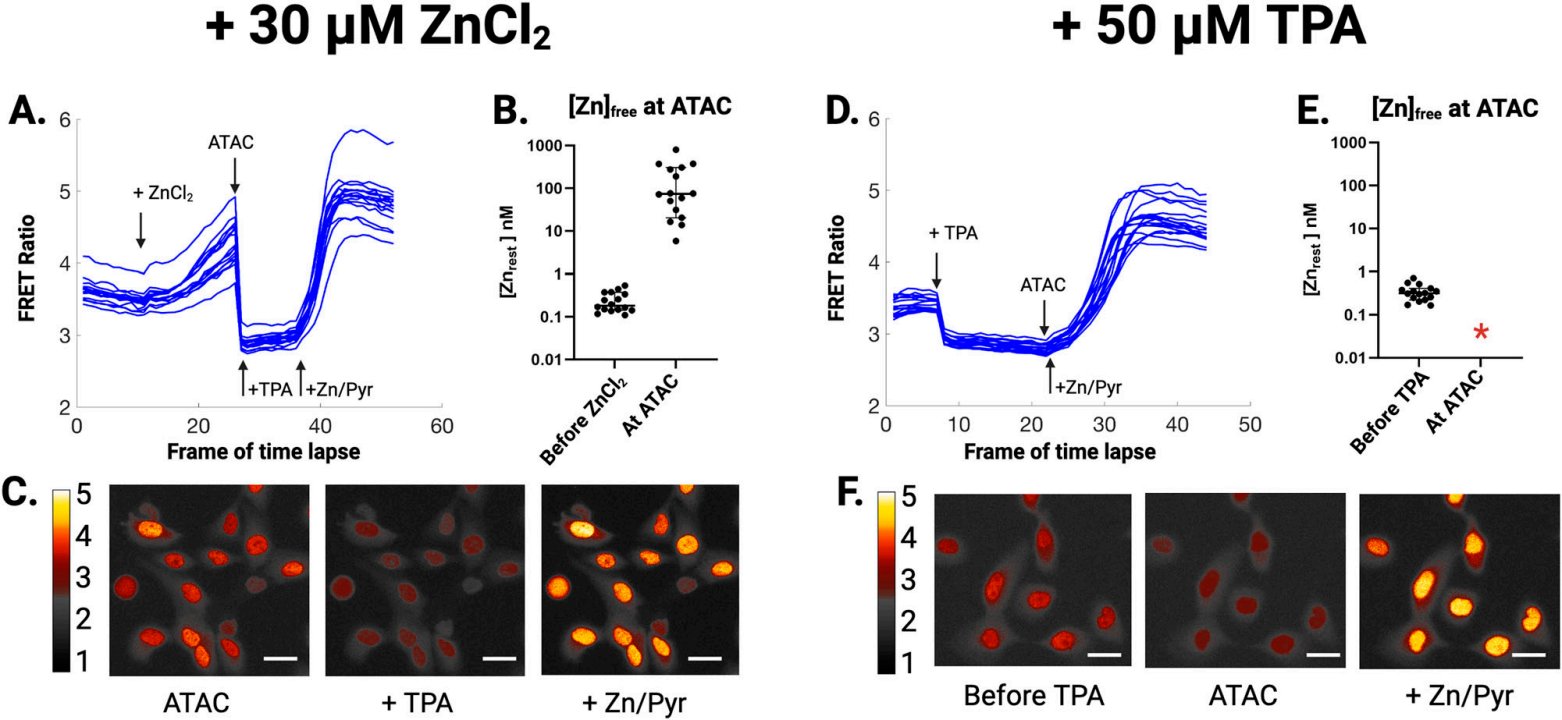
MCF10A cells are susceptible to perturbations in nuclear Zn^2+^. **(A)** Background-corrected FRET ratio traces for MCF10A cells expressing NLS-ZapCV2. Addition of ZnCl_2_ for 30 min results in an increase in labile Zn^2+^ from 150 pM to 75 nM. Addition of the Zn^2+^ chelator TPA followed by the addition of Zn^2+^ and pyrithione at 75 min allows for calibration of the sensor and subsequent quantification of labile Zn^2+^. **(B)** Quantification of Zn^2+^ at rest and at the time point of ATAC-seq. **(C)** Pseudo-colored ratio images of representative cells at the indicated points. **(D)** FRET ratio traces of MCF10A cells treated with TPA to deplete labile Zn^2+^, followed by calibration. **(E)** Quantification of Zn^2+^ at rest. The asterisk indicates that Zn^2+^ cannot be accurately quantified because it is at the lower detection limit of the sensor. Zn^2+^ is estimated to be ∼1 pM. **(F)** Pseudo-colored ratio images of representative cells at the indicated points. Each trace represents a single cell in the field of view. Lookup table values refer to the FRET ratio (background-corrected FRET channel/background-corrected CFP channel). Scale bar = 20 μm.

To determine which TFs may be activated in Zn^2+^ replete or Zn^2+^ deficient conditions, we performed ATAC-seq on MCF10A cells subjected to perturbations of Zn^2+^. We first used a genetically encoded FRET sensor (NLS-ZapCV2) (Fiedler et al, 2017) that is specific for measuring labile (freely exchangeable) Zn^2+^ in the nucleus. Addition of 30 μM ZnCl_2_ to the extracellular media caused a relatively slow but measurable rise in labile Zn^2+^ (Fig 1A–C). To limit any secondary effects that may occur from downstream activation of TFs, we opted to collect cells for ATAC-seq after 30 min of ZnCl_2_ treatment. In situ calibration of the sensor combined with the binding parameters (K_D_’ = 5.3 nM, n = 0.29 [Sanford et al, 2019]) revealed that at 30 min there was a change in labile Zn^2+^ from 150 pM to 75 nM. Addition of 50 μM of the Zn^2+^ chelator tris(2-pyridylmethyl)amine (TPA) resulted in a rapid decrease in nuclear Zn^2+^ to less than 1 pM (the minimum concentration of labile Zn^2+^ that can be quantified using NLS-ZapCV2) (Fig 1D–F). To keep treatments consistent, we also treated cells for 30 min with 50 μM TPA.

**Figure 2.**
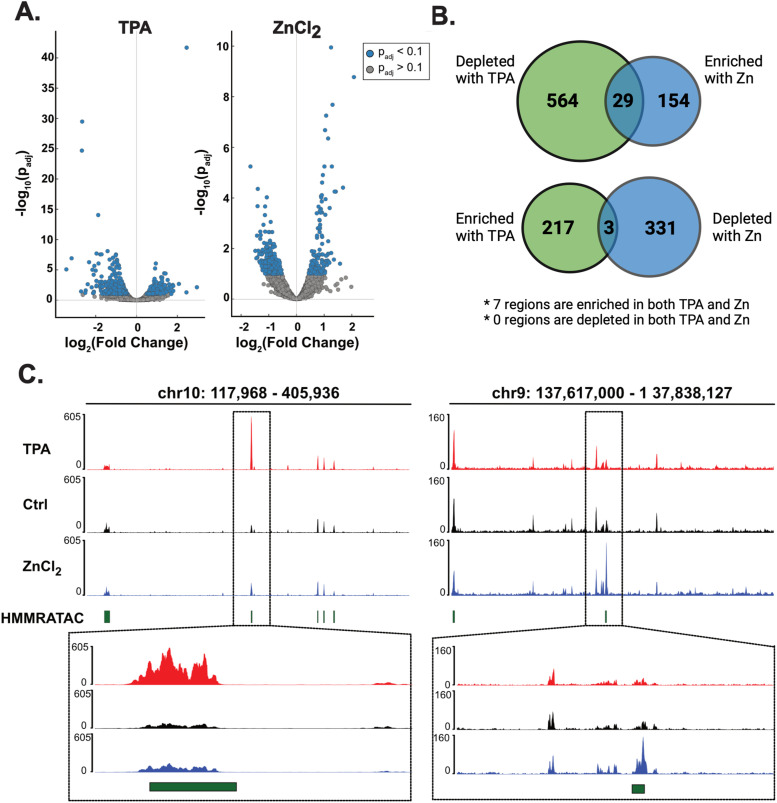
Perturbing cellular Zn^2+^ causes global changes in chromatin accessibility. **(A)** Volcano plots depicting genomic regions that exhibit a change in chromatin accessibility for cells treated with 50 μM TPA (left) or 30 μM ZnCl_2_ (right). A positive log_2_FoldChange indicates an increase in accessibility, whereas a negative log_2_FoldChange indicates a decrease in accessibility. **(B)** DESeq2 differential accessibility analysis shows that most peaks are uniquely accessible depending on Zn^2+^ status. The overlap in the Venn diagrams indicates the peaks which are inversely accessible between the denoted treatments. **(C)** Genomic tracks showing the top hit for differential accessibility for TPA (left) and ZnCl_2_ (right) treated cells. Green boxes denote accessible chromatin as annotated using HMMRATAC. Source data are available for this figure.

**Figure 3.**
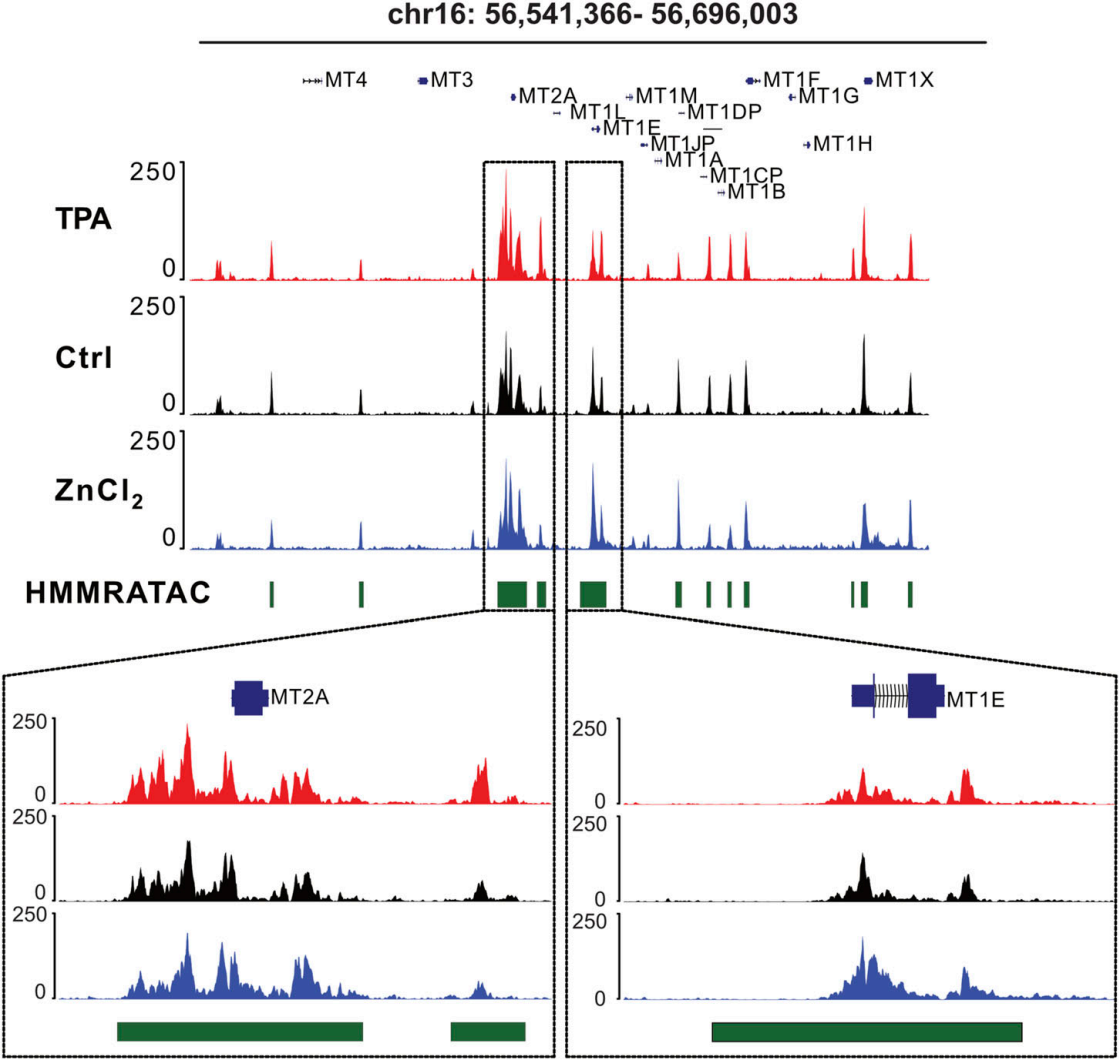
Genomic regions encompassing the Zn^2+^ regulatory genes *MT2A* and *MT1E* show increased accessibility with ZnCl_2_ treatment. Top, the ∼154 kb region of chromosome 16 where all *MT* isoforms reside. Bottom, zoomed in views of the *MT2A* locus (left) and the *MT1E* locus (right).

We performed ATAC-seq to assess which regions in the genome are differentially accessible in Zn^2+^ deficient and Zn^2+^ replete states. Treatment with TPA or ZnCl_2_ induced broad changes in global chromatin accessibility (Fig 2A). TPA treatment impacted accessibility more than ZnCl_2_ treatment (813 versus 517 differentially accessible regions with TPA versus ZnCl_2_) (Fig 2B, SdataF2). Fig 2C shows genomic tracks for the top hit for TPA and ZnCl_2_ treated cells. In both treatments, there was an overall trend towards reduced accessibility, with 593 regions and 334 regions having negative log_2_FoldChanges, corresponding to 73% and 64% of the differentially accessible regions for TPA and ZnCl_2_, respectively. Interestingly, whereas treatment with TPA led to broad changes in accessibility, suggestive of non-specific global decreases in accessibility, elevated Zn^2+^ led to a smaller number of highly significant changes in accessibility in select regions, suggesting that perhaps elevation of Zn^2+^ leads to changes in specific regions of chromatin (Fig 2A). One of the top hits showing increased accessibility with ZnCl_2_ treatment was a region (chr16:56623012–56628480; log_2_FC = 1.14, *P*_adj_ = 4.47 × 10^−7^) directly aligned with the metallothionein isoform *MT1E*, a gene regulated by the MTF1 in response to increased levels of Zn^2+^ (Fig 3). In addition, the region overlapping a second metallothionein isoform, *MT2A*, also showed increased accessibility though to a lesser extent (chr16:56605751–56611835; log_2_FoldChange = 0.611, *P*_adj_ = 0.018). These results show that perturbations of the labile Zn^2+^ pool can alter the landscape of accessible chromatin in a short (30 min) time frame, with Zn^2+^ depletion leading to global decreases in chromatin accessibility and Zn^2+^ increases leading to more specific increases in accessibility. Furthermore, we observe changes in accessibility in genomic regions corresponding to key zinc regulatory genes (*MT1E* and *MT2A*), as would be expected upon perturbation of metal homeostasis.

### TFEA reveals differential enrichment of TF motifs with open chromatin

To determine which TFs may be activated or repressed in each treatment, we performed TFEA (Rubin et al, 2021). Briefly, regions of open chromatin were annotated as peaks using HMMRATAC (Tarbell & Liu, 2019) and peaks from two technical replicates were combined using the muMerge algorithm (Rubin et al, 2021). Then, each of 1279 TF motifs from HOCOMOCO (Kulakovskiy et al, 2018) were subjected to motif scanning using Find Individual Motif Occurrences (FIMO) to identify potential TF-binding motifs within the detected peaks (Grant et al, 2011), and differential expression analysis using DESeq2 was performed to generate a ranked list of peaks that were differentially accessible between control and treatment conditions. This was used to generate an enrichment score (E-score) to determine which TF motifs were differentially enriched in regions of newly open chromatin between the control and treatment conditions. For this analysis we separated promoter regions, defined as 1,000 bp upstream of transcription start sites from distal regulatory regions, defined as regions more than 1,000 bp from a transcription start site.

**Figure 4.**
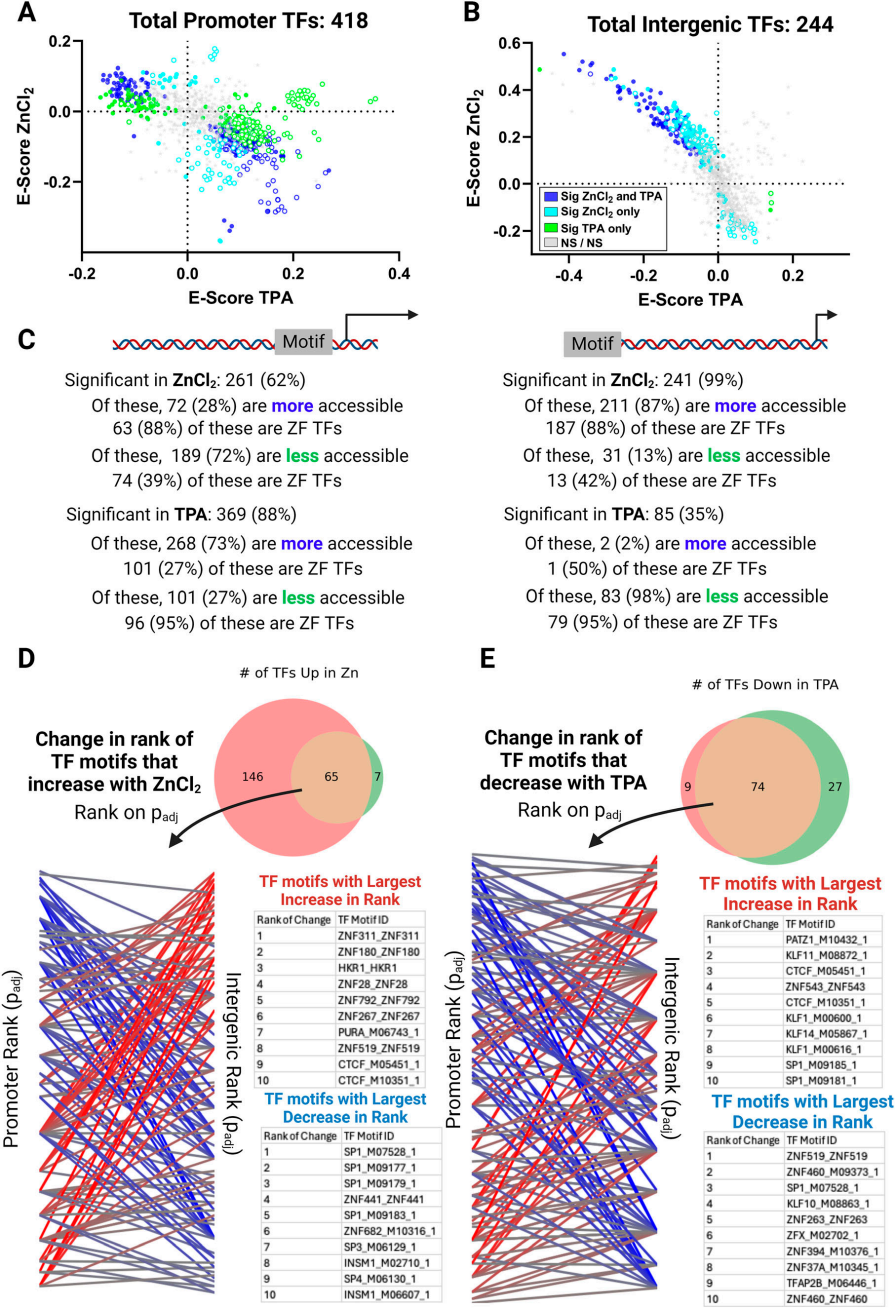
Perturbing cellular Zn^2+^ causes changes in TF motif enrichment that differ at promoters versus distal regulatory regions. **(A, B)** Enrichment plot at promoters (A) and distal regulatory regions (B) showing differential enrichment in ZnCl_2_ versus TPA. Dark blue = differentially enriched in both ZnCl_2_ and TPA, light blue = differentially enriched in ZnCl_2_, green = differentially enriched in TPA. Closed circles are ZF TFs. Open circles are not ZF TFs. **(C)** Summary of the changes. **(D)** Analysis of the motifs that are differentially enriched in ZnCl_2_ in both intergenic (pink) and promoter (green) region. **(E)** Analysis of the motifs that are differentially enriched in TPA in both intergenic (pink) and promoter (green) region. Overlapping motifs were ranked by their *P*_adj_ values. Source data are available for this figure.

Perturbation of Zn^2+^ led to many changes in the enrichment of TF motifs in regions of open chromatin. We found 685 motifs were differentially enriched with a *P*_adj_ < 1 × 10^−7^, corresponding to 507 unique TFs, 434 of which were zinc-finger TFs. The pattern of perturbation and the types of TFs that were most significantly affected were notably different at promoters compared with distal regulatory regions. Fig 4 shows the enrichment score in ZnCl_2_ (elevation of labile Zn^2+^ to 75 nM) compared with the enrichment score in TPA (depletion of labile Zn^2+^ to ∼1 pM) for individual TFs in either promoter regions (Fig 4A, SdataF4.1) or intergenic regulatory regions (Fig 4B, SdataF4.2). Strikingly, in distal regulatory regions, a large number of TFs show significant motif enrichment when chromatin becomes more open in response to elevated Zn^2+^ (211 TFs, 187 of which are zinc-finger TFs). 83 of these TFs show motif depletion in regions of open chromatin in low zinc, suggesting reciprocal activation of these TFs in response to Zn^2+^ perturbation. In contrast to the intergenic region, at promoters the changes are dominated by TFs whose motifs are enriched in regions of open chromatin upon Zn^2+^ depletion. 268 of the 418 TFs with motifs that are differentially enriched in promoter regions with *P*_adj_ < 1 × 10^−7^ show enrichment in TPA. 158 of these TFs are also depleted in high Zn^2+^, suggesting reciprocal activation upon zinc perturbation. Notably, zinc-finger TFs (ZF TFs) are under-represented in this group (101 of 268 or 38%, compared with the 48% of the human TFs that are ZF TFs), suggesting that differential enrichment of these TF motifs may result from global changes in chromatin organization in low Zn^2+^. Fig 4C summarizes the differential enrichment results at promoters and intergenic regions, showing that a given Zn^2+^ perturbation tends to have an opposite effect in each region (e.g., elevation of Zn^2+^ causes enrichment of motifs with open chromatin in intergenic regions and depletion of such motifs at promoters).

Elevation of zinc has the biggest effect on chromatin around TF motifs at distal regulatory regions whereas depletion of zinc has the biggest effect on chromatin around TF motifs at promoters. There were a significant number of motifs (and unique TFs) that were differentially enriched in both promoter regions and intergenic regions, however there was a notable shift in the relative significance of these motifs. Fig 4D shows the relative significance (*P*_adj_ ranked from high to low) for TF motifs that were differentially enriched at both promoters and intergenic regions in high Zn^2+^. The plot reveals major changes in the rank order of TF motifs that were enriched in high Zn^2+^. Fig 4E shows a similar comparison for overlapping TF motifs that were depleted in low Zn^2+^. These results reveal that perturbation of Zn^2+^ affects TF motif enrichment differently at promoters versus distal regulatory regions.

We noticed that a large number of the differentially enriched motifs corresponded to Krüppel-type zinc finger (ZNF) TFs. ZNF proteins contain a median of 12 zinc fingers, suggesting they bind relatively long and specific sequences of DNA. Still, they are largely uncharacterized. A comprehensive catalog of ZNF proteins found that one third of all ZNF TFs contain a Krüppel associated box (KRAB) domain which recruits histone deacetylase proteins giving rise to transcriptional repression (Huntley et al, 2006). Given that we found 86% of the differentially enriched TFs in intergenic regions were ZF TFs, we examined what fraction of these also contained KRAB domains. We found that 97 TFs (40%) contain KRAB domains, suggesting this class of TFs is slightly overrepresented in the repertoire of TFs that bind in distal regulatory regions predicted to be differentially accessible by zinc.

As noted above, many of the TF motifs that emerge from TFEA correspond to TFs that either directly bind Zn^2+^ or are known to be correlated with metal homeostasis. That elevation of Zn^2+^ led to enrichment of motifs for zinc-finger TFs in regions of open chromatin in intergenic regions suggests that the functionality of zinc-finger TFs may be sensitive to increases in the labile Zn^2+^ pool in the nM range. On the other hand, functional annotation of TF motifs that are enriched in open chromatin in TPA did not reveal any meaningful trends, suggesting that perhaps these conditions lead to non-specific global changes in chromatin accessibility. For the motifs that are enriched in elevated Zn^2+^ and depleted in Zn^2+^ deficiency, many correspond to poorly annotated Zn^2+^ finger proteins, such as ZNFs and ZSCANs. However, there were some hits that are well characterized and that were particularly intriguing. For example, the CCCTC-binding factor (CTCF) is an 11-zinc-finger protein involved in chromatin organization. Two CTCF motifs were globally enriched in elevated Zn^2+^ (E-score = 0.44 and 0.38, *P*_adj_ = 1 × 10^−197^ and 1 × 10^−176^) and depleted in TPA (E-score = −0.29 and −0.23, *P*_adj_ = 1 × 10^−71^ and 1 × 10^−64^) in intergenic regions. Our laboratory recently used single molecule microscopy to show that CTCF senses low Zn^2+^ conditions and becomes significantly more mobile when Zn^2+^ is depleted, suggesting decreased association with chromatin (Damon et al, 2022). We also observed that a motif of the zinc-finger DNA methyltransferase DNMT1 was strongly enriched in elevated Zn^2+^ (E-score = 0.52, *P*_adj_ = 1 × 10^−56^) and depleted in TPA (E-score = −0.36, *P*_adj_ = 1 × 10^−29^) in intergenic regions. DNMT1 is not a canonical TF but methylates DNA during DNA synthesis, and our laboratory has previously shown that DNA synthesis is impaired in Zn^2+^ deficiency (Lo et al, 2020).

### Correlation of TFEA enrichment with TF occupancy for the candidate TF p53

**Figure 5.**
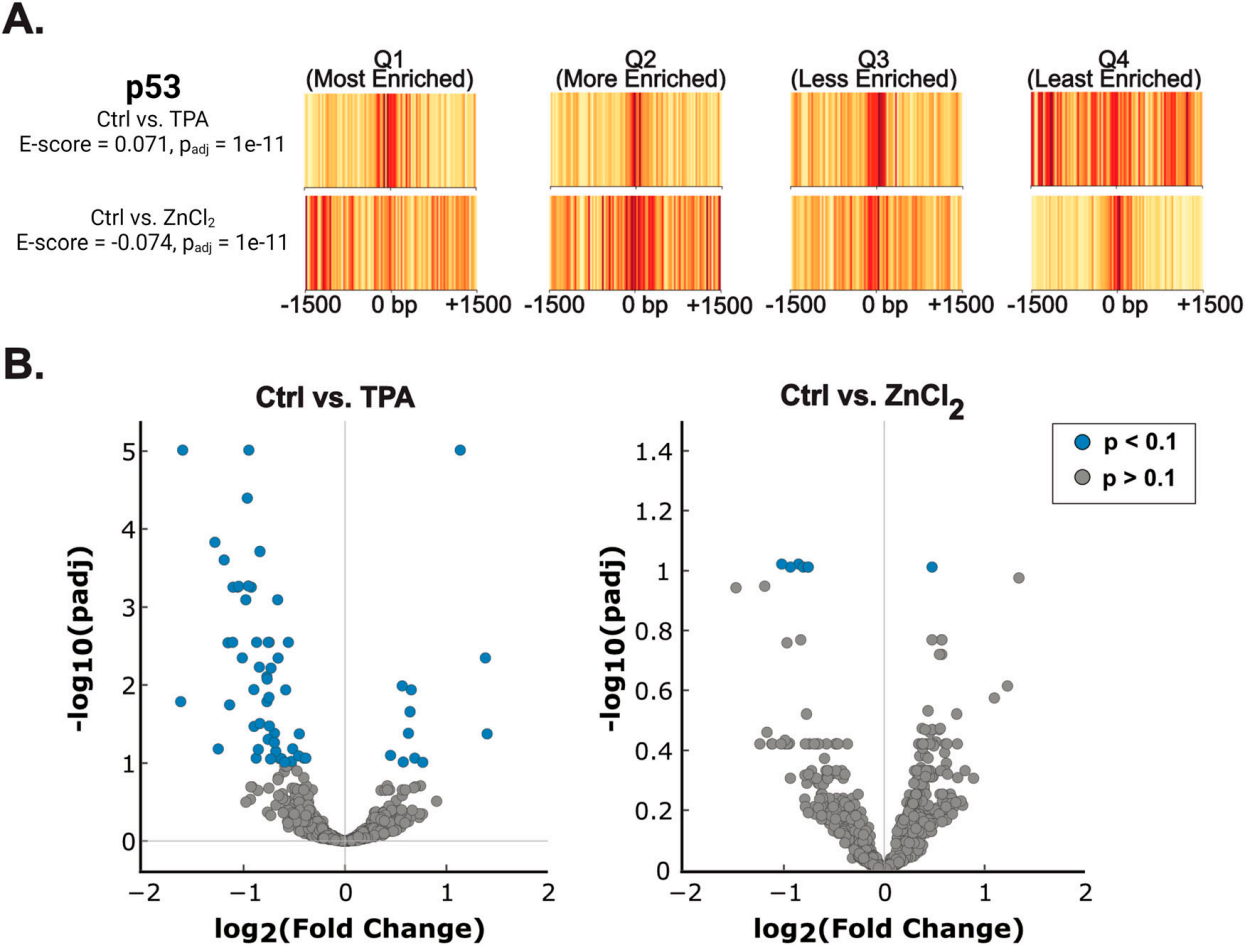
p53 motifs show differential enrichment upon Zn^2+^ perturbation. **(A)** Barcode plots that show enrichment of the p*53* motif upon treatment of MCF10A cells with TPA (top). Addition of exogenous ZnCl_2_ results in depletion of the same motif (bottom). The barcode plots represent each quartile (Q1–Q4) of the enrichment curves generated for the p53 motif. Red indicates more enrichment of the motif; yellow is less enrichment of the motif. **(B)** A subset of p53 binding sites from a ChIP-seq dataset (GSM3378513) are differentially accessible depending on cellular Zn^2+^ status. ATAC-seq reads were mapped to 2,164 ChIP-seq peaks and subjected to differential accessibility analysis using DESeq2. With TPA treatment, 62 peaks showed significant (*P*_adj_ ≤ 0.1) changes in accessibility (51 decreased, 11 increased). With ZnCl_2_ treatment, seven peaks showed significant changes in accessibility (one increased, five decreased). Source data are available for this figure.

Whereas TFEA identifies TF motifs that become enriched in newly opened chromatin upon a given treatment, it does not directly reveal whether TF occupancy on DNA has changed. To probe how TFEA enrichments correlate with TF occupancy, we selected a candidate TF and used chromatin immunoprecipitation to pull down regions of DNA bound to the TF, followed by quantitative real-time PCR (ChIP-qPCR) to quantify how much of the TF is bound to a given target gene. Our two main goals were to determine whether differential enrichment identified by TFEA could be used to predict changes in TF occupancy and to evaluate a bioinformatic plus ChIP-qPCR workflow that could be used to validate TFEA predictions. As a TF target, we selected p53 (encoded by the *TP53* gene) because of its differential motif enrichment (Fig 5A) and because there are multiple ChIP/ChIP-seq datasets indicating robust vetting of the antibody for ChIP.

Because ATAC-seq identified more than 50,000 peaks, we sought to narrow the number of possible p53 binding sites against which to perform ChIP-qPCR. Hence, we used a previously published p53 ChIP-seq dataset (GSM3378513) (Karsli Uzunbas et al, 2019) where MCF10A cells were treated with 5 μM Nutlin-3A for 6 h to activate p53. We reasoned that this ChIP-seq dataset would allow us to identify bona fide regions of the genome to which p53 binds. Furthermore, we intersected the ChIP-seq dataset with a PRO-seq dataset (GSE227931) where MCF10A cells were treated with Nutlin-3A (10 μM, 3 h) to identify regions of active transcription marked by the presence of bidirectional transcripts (Azofeifa & Dowell, 2017). This reduced the number of potential p53 binding sites from approximately 13,500 (as identified in the ChIP-seq dataset) to 2,164 (ChIP-seq intersected with PRO-seq). We then took this list and queried whether any of the validated p53 binding sites were differentially accessible in response to Zn^2+^ in our ATAC-seq dataset (Fig 5A). Differential accessibility analysis using DESeq2 revealed that with TPA treatment, 62 sites were differentially accessible (*P*_adj_ ≤ 0.1), with 51 of these being less accessible and 11 of these becoming more accessible (Fig 5B, Table S1, SdataF5). Conversely, treatment with ZnCl_2_ resulted in six regions being differentially accessible, with five of these being less accessible and one being more accessible (Fig 5B, Table S2, SdataF5). No sites with significant changes in accessibility were shared between both treatments.

Table S1. p53 ChIP sites that showed significant (*P*_adj_ ≤ 0.1) changes in accessibility with TPA treatment. ChIP location denotes the genomic coordinates (hg38) of the ChIP peak. Peaks are sorted by ascending −log_10_(*P*_adj_). Base mean is defined as the average of the normalized count values, dividing by size factors, taken over all samples.

Table S2. p53 ChIP sites that showed significant (*P*_adj_ ≤ 0.1) changes in accessibility with ZnCl_2_ treatment, quantified by DESeq2. ChIP location denotes the genomic coordinates (hg38) of the ChIP peak. Peaks are sorted by ascending −log_10_(*P*_adj_). Base mean is defined as the average of the normalized count values, dividing by size factors, taken over all samples.

**Figure 6.**
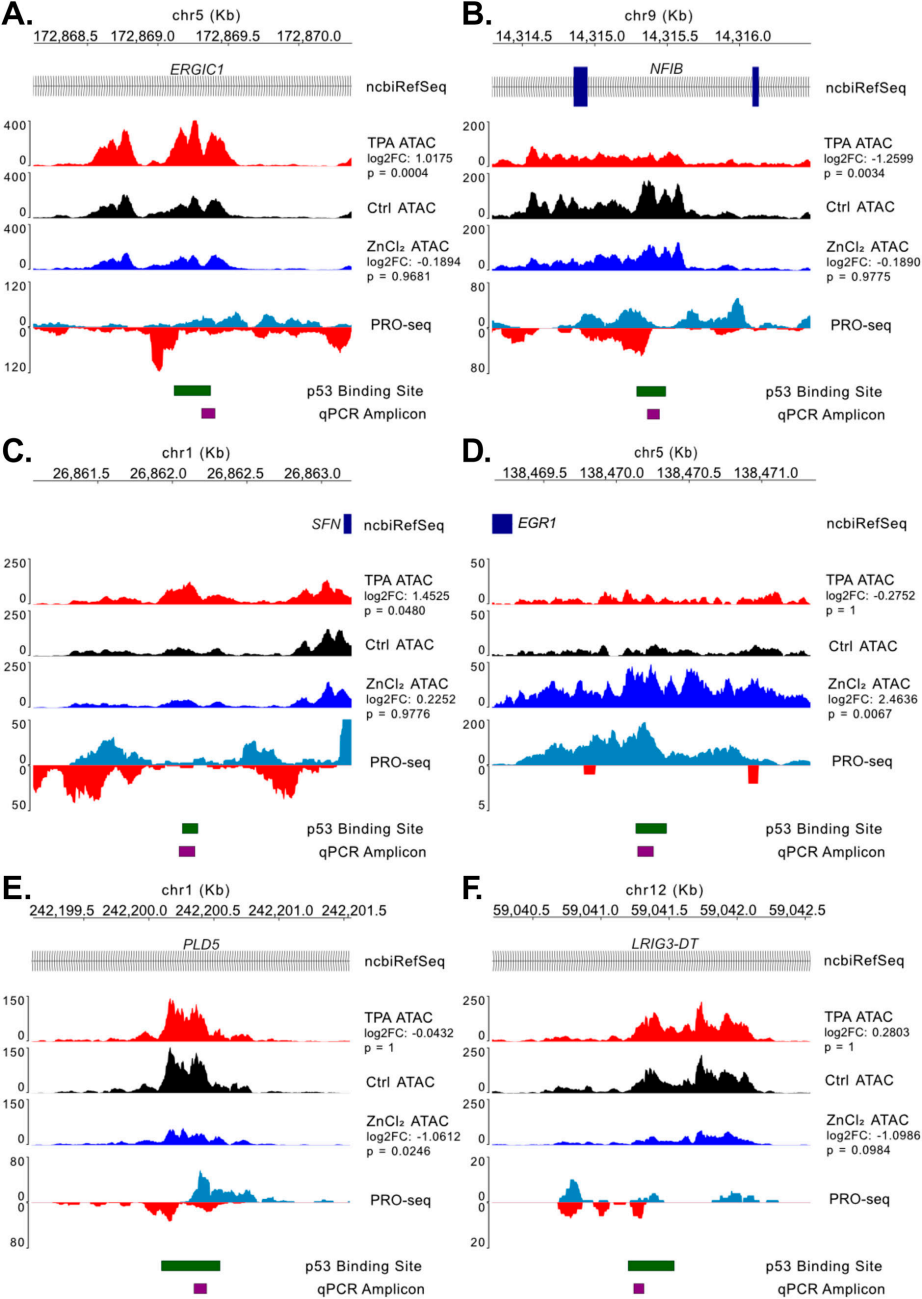
Selected regions of interest for ChIP-qPCR assays with ATAC-seq coverage tracks for MCF10A cells treated with either 50 μM TPA, a media-only Control (Ctrl), or 30 μM ZnCl_2_. Also shown are the PRO-seq coverage tracks for MCF10A cells treated with 10 μM Nutlin-3A for 3 h (GSE227931), the annotated region from the GSM3378513 p53 Nutlin-3A ChIP-seq dataset, and the predicted amplicon from ChIP-qPCR. **(A, B, C, D, E, F)** Coverage tracks as noted above for the (A) ERGIC1, (B) NFIB, (C) SFN, (D) EGR1, (E) PLD5, and (F) LRIG3-DT regions.

From this list of differentially accessible sites, we selected six p53 binding sites against which to perform ChIP-qPCR. As shown in Fig 6, the six target sites (*ERGIC1*, *NFIB*, *SFN*, *EGR1*, *PLD5*, *LRIG3-DT*, Fig 6A–F, respectively) were selected because they showed differential chromatin accessibility via ATAC-seq for at least one of the Zn^2+^ perturbations and were associated with active transcription in MCF10A cells in response to Nutlin-3A based on PRO-seq bidirectional tracks (Fig 6). We also included the well-established p53 target gene *CDKN1A* (p21) as a positive control for immunoprecipitation and a negative control primer set that would not be expected to show enrichment with and without the p53 antibody.

**Figure 7.**
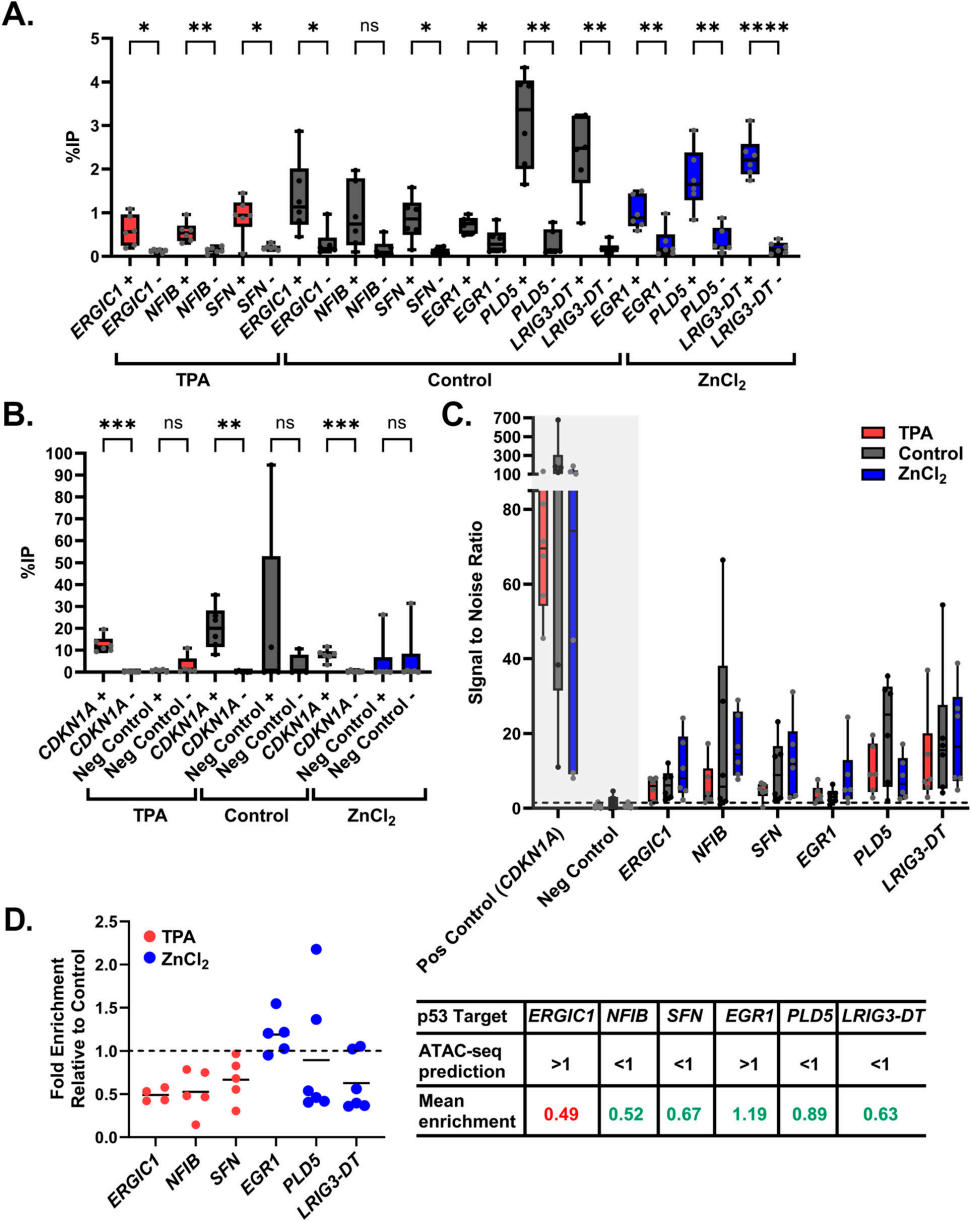
ChIP-qPCR results for putative p53 targets. **(A)** %IP of each p53 target from ChIP-qPCR samples incubated with (+) or without (−) p53 antibody. Each data point is a biological replicate averaged between two technical replicates. **(A, B)** Same as (A) but for the negative and positive qPCR controls. **(C)** Signal-to-noise ratio of each ChIP-qPCR p53 target. Dotted line denotes a SNR value of 1.5. Controls for p53 binding are shown in the gray box. **(D)** Fold enrichment relative to the control for each ChIP-qPCR p53 target (left). Dashed line indicates enrichment ratio of 1 (no enrichment), and lines within each dataset represent the mean. Comparison between the ATAC-seq prediction of increased or decreased accessibility (>1 or <1, respectively) and experimental fold enrichment for each gene target by ChIP-qPCR (right).

We performed ChIP-qPCR against the target regions using a p53 specific antibody. We incubated chromatin with Protein A/G beads to assess the overall background signal and calculated the %IP of samples incubated for each p53 target (Fig 7A) and for the positive and negative qPCR controls (Fig 7B). As expected, there was a significant increase in the %IP for the +p53 antibody samples compared with the −p53 antibody samples. The signal to noise ratio (SNR) was calculated by dividing the %IP of the +p53 antibody sample by the %IP of the beads-only (−p53) sample for each target (Fig 7C). Of our gene targets, *CDKN1A* (p21) had the strongest overall signal and SNR, as expected given that it is a high affinity p53 target. The negative control (Negative Control Primer Set 1) showed no difference between the plus and minus antibody condition (Fig 7B), further validating the ChIP conditions. Most gene targets had comparable signal and SNR across conditions (average SNR > 5), with ZnCl_2_ samples often displaying the largest SNR value except for *NFIB* and *PLD5*, which had larger SNR values in the control (no zinc treatment) samples. *EGR1* showed the lowest overall SNR across all conditions, with the TPA and control conditions displaying an average value lower than five (TPA: 3.52; Control: 3.24; ZnCl_2_: 7.91).

The fold enrichment of p53 binding in our TPA and ZnCl_2_ treatments relative to the control was calculated by dividing the %IP of the +p53 treated (TPA or ZnCl_2_) ChIP samples by the +p53 of untreated (Control) ChIP samples for each target. The selected gene targets showed changes in p53 binding as a consequence of Zn^2+^ perturbation, where the fold enrichment values (indicative of increased or decreased binding) largely agreed with their respective changes in accessibility (for accessibility changes where *P*_adj_ < 0.1). Fig 7D shows the fold enrichment ratio for each of the six targets along with the ATAC-seq prediction of increased or decreased accessibility (>1 or < 1, respectively). For five of the six targets, the mean enrichment ratio generally agreed with the accessibility predicted by ATAC-seq. *ERGIC1* was the one target gene where differential p53 binding was opposite the accessibility changes by ATAC-seq. In TPA, accessibility was increased (Fig 6A), whereas binding by p53 decreased (0.49 versus 1 in TPA versus control). Overall, our results indicate that Zn^2+^-induced changes in the enrichment of TF motifs in regions of open chromatin can be associated with changes in TF binding to target genes.

## Discussion

It has long been known that metal homeostasis is essential for proper cellular and organismal health, but relatively little has been performed to probe the genomic landscape when this homeostasis is disrupted. Here, we performed ATAC-seq on MCF10A mammary epithelial cells following treatments that either raised intracellular Zn^2+^ to approximately 75 nM or depleted intracellular Zn^2+^ to less than 1 pM. In both Zn^2+^ rich and Zn^2+^ deficient states, we saw global changes in chromatin accessibility associated with differential enrichment of numerous TF motifs, with notable differences in the pattern of enrichment in promoter regions versus distal regulatory regions. Importantly, the Zn^2+^ perturbations used here are subtle and within the range of Zn^2+^ dynamics observed during normal physiology. For example, we recently showed that there is a pulse of labile Zn^2+^ immediately following mitosis in the mammalian cell cycle where Zn^2+^ increases to ∼1.6 nM for a few hours (Rakshit et al, 2023). The results presented here suggest that dynamics in labile Zn^2+^ can alter chromatin accessibility, leading to potential changes in transcriptional regulation.

The pattern of perturbation and the types of TFs that were most significantly affected were notably different at promoters compared with distal regulatory regions. In particular, we found that the dominant change in distal regulatory regions was that elevation of Zn^2+^ led to strong enrichment of TF motifs with open chromatin, consistent with an increase in activity of the associated TFs. Most of these TFs (88%) were zinc-finger TFs, and 39% also showed depletion in low zinc, suggesting reciprocal activation by zinc perturbations. Conversely, promoter regions were more strongly influenced by Zn^2+^ depletion, where TPA treatment led to enrichment of TF motifs with open chromatin. Zinc-finger TFs were under-represented in this population (38%).

Many of the differentially enriched motifs, particularly in intergenic regions, are for TFs in the ZNF, ZSCAN, or ZBTB families. The majority of these TFs are poorly characterized beyond the knowledge that they contain Zn^2+^ binding domains and may be involved in transcriptional regulation. However, a recent study found that evolutionarily conserved Zn^2+^ finger proteins were more apt to bind to promoters whereas Zn^2+^ finger TFs that evolved more recently were more likely to bind to transposable elements found in intergenic regions (Imbeault et al, 2017). Transposable elements have long been thought of as invaders that cause genomic instability and disease (Hancks & Kazazian, 2010; Jacobs et al, 2014), but more recently they have been recharacterized as potential hubs of transcriptional regulation, as on average 20% of all binding sites in TF ChIP-seq datasets colocalize with transposable elements (Sundaram et al, 2014). It is therefore possible that Zn^2+^ finger TFs regulate these sites, and it is most likely in a repressive role as work has shown that deletion of clusters of Zn^2+^ fingers in mice reactivates retrotransposons (Wolf et al, 2020). Indeed, we found that ZF TFs dominated differential enrichment in distal regulatory regions and 40% of these TFs contain a KRAB domain, responsible for transcriptional repression. The most significant intergenic hit was the chromatin remodeling protein, CTCF, where two CTCF motifs were depleted in low Zn^2+^ and enriched in elevated Zn^2+^. This is consistent with a recent study from our lab indicating that upon Zn^2+^ decrease, CTCF is more mobile with a decreased residence time on DNA, suggesting decreased association with chromatin (Damon et al, 2022).

Although we found that perturbing the labile Zn^2+^ pool leads to significant changes in the accessibility of many genomic sites, the mechanism leading to these changes has not been identified. At any given time, there are thousands of zinc-coordinating TFs in the nucleus, and a change in labile zinc could globally affect the function of these TFs. In addition, there are numerous chromatin-modifying enzymes such as histone deacetylases, acetyltransferases, and methyltransferases, some of which contain a zinc-binding site. We imagine that there are at least three possible non mutually exclusive mechanisms that could give rise to the observed changes. First, for zinc-finger TFs whose motifs are enriched in elevated Zn^2+^ and depleted in low Zn^2+^, zinc-occupancy, and hence DNA-binding ability, of these TFs may be directly influenced by the labile Zn^2+^ pool. Consistent with this possibility, we previously found that low Zn^2+^ causes increased mobility and decreased dwell-time of CTCF in the nucleus, suggesting that in low Zn^2+^ CTCF’s ability to interact with chromatin is decreased (Damon et al, 2022). Second, Zn^2+^ may indirectly affect TF function via cell signaling. For example, our group has found that elevated Zn^2+^ leads to the activation of the MAPK pathway, and hence activation of Elk1, CREB, p90Rsk, and cJun (Anson et al, 2021). Third, many zinc-finger TFs and zinc-dependent enzymes directly modulate chromatin. Zn^2+^ finger TFs such as ZBTB33 are known to associate with methylated DNA, a mark of heterochromatin and gene repression (Hodges et al, 2020; Yusuf et al, 2021), and the primary DNA methyltransferase that preserves methylation during cell division, DNMT1, has a Zn^2+^ binding domain that aids in its recognition of hemimethylated DNA (Ren et al, 2018). Whereas the treatment window of our work was likely too short to alter DNA methylation patterns, it is possible that chronic disruption of Zn^2+^ homeostasis could perturb DNA methylation patterns, as was recently shown in *Arabidopsis* (Chen et al, 2018). As noted previously, CTCF is a chromatin organizing protein with 11 Zn^2+^ finger motifs that plays an important role in insulating topologically associated domains (TADs) of chromatin (Dixon et al, 2012). Changes to the labile Zn^2+^ pool that affect CTCF’s function could disrupt TADs, as it was shown that CTCF degradation in mouse embryonic stem cells distorts TAD architecture (Nora et al, 2017), rendering potential changes to chromatin accessibility. Given the diversity of zinc-binding proteins, and the fact that we see different patterns of change at promoters versus intergenic regions, it is likely multiple mechanisms contribute to the observations in this work.

Here, we used TFEA to couple differential accessibility with TF motif scanning to infer global activation or repression of TFs. Positive enrichment of a TF motif does not necessarily indicate that all the motif occurrences are bona fide TF-binding sites, nor does it indicate that all TF-binding sites will show an increase in accessibility. To develop a pipeline for validating TFEA predictions, we used publicly available datasets to narrow the scope. For example, TFEA applied to our ATAC-seq dataset detected > 5,700 p53 motifs. We used a published p53 ChIP-seq dataset to filter these hits to genomic regions where p53 had previously been shown to bind. We further applied a p53 PRO-seq dataset to identify p53 binding sites near regions of active transcription, as identified by bidirectional transcripts. This analysis revealed only ∼2,100 of the ChIP-seq binding sites showed productive transcription upon activation of p53 by a known agonist (Nutlin-3A). Of these ∼2,100 binding sites, 86 showed differential accessibility in response to cellular Zn^2+^ perturbation. From this narrowed list, we selected six putative p53 binding sites to query with ChIP-qPCR. For five of the six sites, p53 binding correlated with local accessibility, such that a decrease in accessibility led to decreased p53 binding and an increase in accessibility correlated with increased p53 binding. These results suggest that TFEA coupled with ATAC-seq can be used to profile which TFs may be activated upon a given perturbation. With the availability of curated ChIP-seq datasets in repositories such as ENCODE and Cistrome, researchers can perform the simple and relatively inexpensive ATAC-seq, scan for enriched TF motifs using TFEA, computationally correlate changes in accessibility with candidate TF-binding sites found within the literature, and then probe a subset of sites using ChIP-qPCR. In conclusion, this article reveals for the first time that chromatin accessibility and transcription factor binding to DNA can be modulated by subtle changes in the labile zinc pool, suggesting that zinc may serve as a previously unrecognized modulator of transcriptional activity.

## Materials and Methods

### Molecular cloning

PiggyBac-NLS-ZapCV2 was generated by digesting PiggyBac-NES-ZapCV2 (Lo et al, 2020) with EcoRI and SalI. NLS-ZapCV2 was amplified from pcDNA(3.1)-NLS-ZapCV2 with EcoRI (5′ end) and SalI (3′ end) restriction sites using the following primers: ggaattGAATTCGCTTGGTACCGAGCATGCC and gaagcgGTCGAGCCACTGTGCTGGATATCTGCAGAA TTC. The NLS-ZapCV2 insert and PiggyBac-NES-ZapCV2 (Lo et al, 2020) were subsequently digested with EcoRI and SalI, and the NLS-ZapCV2 insert was ligated into the PiggyBac backbone.

### Cell culture

Wildtype MCF10A cells (#CRL-10317; ATCC) were cultured at 5% CO_2_ in DMEM/F12 (#11320033; Thermo Fisher Scientific) supplemented with 5% Horse Serum (#16050122; Thermo Fisher Scientific), 1% penicillin/streptomycin (#15070063; Thermo Fisher Scientific), 20 ng/ml EGF (#PHG0313; Thermo Fisher Scientific), 0.5 mg/ml hydrocortisone (#H0888; Sigma-Aldrich), 100 ng/ml Cholera toxin (#C8052; Sigma-Aldrich, and 10 μg/ml insulin (#12585014; Thermo Fisher Scientific). To generate MCF10A cells stably expressing PiggyBac-NLS-ZapCV2, ∼1.5 million wildtype MCF10A cells were incubated with 1 μg PiggyBac-NLS-ZapCV2 and 200 ng Super PiggyBac Transposase (#PB210PA-1; Systems Biosciences) and electroporated using a Neon Electroporation System (Thermo Fisher Scientific). Cells were plated into antibiotic-free media (the same composition as above, minus antibiotic) for 24 h and then underwent puromycin selection using 0.5 μg/ml puromycin for 7 d before experiments.

### Live cell fluorescence microscopy

FRET sensor calibrations were performed on a Nikon widefield microscope equipped with a Ti-E perfect focus system, an iXon3 EMCCD camera (Andor), mercury arc lamp, Lambda 10-3 filter changer (Sutter Instruments), and a 20x Plan Apo air objective (Nikon). Filter settings used were: CFP (434/16 excitation, 458 dichroic, 470/24 emission), YFP FRET (434/16 excitation, 458 dichroic, 535/20 emission), and YFP (495/10 excitation, 515 dichroic, 535/20 emission). Camera exposure times of 300 ms, EM multiplier of 300, and 1 MHz readout speeds were consistent across all channels.

### Quantification of nuclear Zn^2+^ levels

MCF10A cells stably expressing PiggyBac-NLS-ZapCV2 were equilibrated at 37°C, 0% CO_2_ in phosphate-free HEPES-buffered Hanks balanced salt solution (HHBSS) containing 1.26 mM CaCl_2_, 1.1 mM MgCl_2_, 5.4 mM KCl, 20 mM HEPES, and 3 g/liter glucose for 30 min before imaging. Baseline resting FRET ratios (R_rest_) were collected for 10 min, at which point ZnCl_2_ was added to and final media concentration of 30 μM for approximately 50 min. Cells were then washed 3x in phosphate-free HHBSS to remove excess ZnCl_2_ and 50 μM of tris(2-pyridylmethyl)amine (TPA) diluted in phosphate-free HHBSS was added to chelate Zn^2+^ and determine the sensor’s minimum FRET ratio (R_min_). Cells were washed 3x in phosphate-free HHBSS (with no CaCl_2_ or MgCl_2_) to remove excess TPA, and a solution containing 119 nM buffered Zn^2+^ (Tsien & Pozzan, 1989), 1.5 μM pyrithione, and 0.001% wt/vol saponin was then added to determine the maximum FRET ratio of the sensor (R_max_). Images were then processed in MATLAB R2017b using previously published analysis code (Lo et al, 2020; Anson et al, 2021). Briefly, nuclei were computationally detected using the fluorescence signal in the YFP FRET channel and the background-corrected FRET ratio was calculated by dividing the background-corrected acceptor (YFP FRET) signal by the background-corrected donor (CFP) signal. For each cell, average labile Zn^2+^ concentrations were calculated using the equation $\left[{Zn}^{2+}\right]={K_{d}\frac{\left(R-R_{\min}\right)}{\left(R_{\max}-R\right)}}^{1/n}$ with a *K*_*d*_ = 5.3 nM and Hill coefficient (n) = 0.29.

### ATAC-seq sample preparation and sequencing

∼50,000 MCF10A cells were pre-incubated in phosphate-free HHBSS for 30 min at 37°C and 0% CO_2_, followed by treatment with either 50 μM TPA, 30 μM ZnCl_2_, or phosphate-free HHBSS for 30 min. Nuclei isolation and transposition followed the OMNI ATAC-seq protocol as previously reported (Corces et al, 2017) using the Illumina Tagment DNA TDE1 Enzyme & Buffer Kit (#20034197; Illumina). Libraries were generated using Illumina Nextera CD Indexes (#20018708; Illumina) and sequenced in paired-end mode (2 × 75 bp) on an Illumina Nextseq 500 at the BioFrontiers Next Generation Sequencing Facility (CU Boulder). ATAC-seq data were collected in two biological replicates for three different conditions: TPA treatment, ZnCl_2_ treatment, and no treatment (control). For differential accessibility analysis, the TPA and ZnCl_2_ treatment conditions were compared to a common control dataset.

### ATAC-seq data processing

Quality control of demultiplexed fastq files was performed using fastQC (v0.11.8) (Babraham Bioinformatics, 2022). Adapters were trimmed using BBDuk (BBDuk Guide, 2022) using the following flags: ktrim = r, qtrim = 10, k = 23, mink = 11, hdist = 1, maq = 10, minlen = 20, tpe, tbo. Resulting trimmed fastq files were mapped in a paired-end fashion with HISAT2 (Kim et al, 2019) (v2.1.0) to hg38. Resulting BAM files were indexed with samtools (v1.8) (Li et al, 2009). Regions of open chromatin were annotated using HMMRATAC (Tarbell & Liu, 2019); for downstream analyses (e.g., differential accessibility, TFEA, etc.), the entire open region, rather than the annotated summit, was used as an input. For assessing which regions of open chromatin were differentially accessible, peaks from all treatments were merged using muMerge (Rubin et al, 2021). Peaks that overlapped with blacklisted genomic regions (Amemiya et al, 2019) were removed using bedtools (v2.28.0). Mapped read counts were then counted using featureCounts in R (v4.0.5) and differential accessibility was determined using DESeq2 (Love et al, 2014).

For assessment of differential TF motif enrichment we used TFEA (Rubin et al, 2021). Within the TFEA pipeline, regions of interest (ROIs) were defined by merging HMMRATAC-called peaks from two biological replicates using muMerge. Read coverages within each ROI were then calculated with HTSeq (v. 0.13.5) and ROIs were ranked according to the significance of differential accessibility with DESeq2 (v. 1.26.0, R v. 3.6.1). For analysis of only promoter or nongenic regions, annotation files were generated from Refseq annotations. Promoters were defined as 1,000 base regions directly upstream from gene transcription start sites, and nongenic annotations were created using bedtools intersect (v. 2.28.0) and included any genomic region outside of a gene or promoter. Ranked differential region lists were then filtered with bedtools to overlap promoter or nongenic annotation files prior to TF motif scanning. TF motif scanning was then performed within the ±1,500 bp window surrounding each ROI center using FIMO (meme v. 5.0.3) FIMO (Grant et al, 2011) at a *P*-value cutoff of 10^−5^ and a curated set of TF position weight matrices (Lambert et al, 2018). TFEA then computed motif enrichment scores for each motif for each treatment relative to the control.

For a single region to be “statically significant” there has to be a very large change in that region. In TFEA, like Gene Set Enrichment Analysis (Subramanian et al, 2005), the assay depends on a ranked list of regions/genes. Therefore, it is not based on the set of significant regions, but on the direction all regions are shifted in differential expression.

### PRO-seq and library preparation methods

MCF10a cells were grown up in DMEM/F12 media with supplements as described (Levandowski et al, 2021) to a confluency of 70% before treatment with Nutlin-3a (10 μM Nutlin-3a in DMSO, #S8059; Selleck) or vehicle (0.1% DMSO) for 3 h before the nuclei were harvested. 10 × 10^6^ nuclei were prepared for nuclear run-on and library preparation as described (Kwak et al, 2013) with modifications (Fant et al, 2020). Samples were sequenced on the Illumina NextSeq 500 platform (single-end × 75 bp). These data are publicly available on NCBI GEO at GSE227931.

### Selection of p53 binding sites for ChIP-qPCR

To find putative p53 binding sites to probe using ChIP-qPCR, we downloaded the BED file containing ChIP-seq peaks from GSM3378513 (Karsli Uzunbas et al, 2019) (p53 ChIP-seq in MCF10A cells treated with Nutlin-3a) from the Cistrome Data Browser (ID #105311; CistromeDB). CistromeDB assesses ChIP-seq datasets on six quality control metrics (sequence quality, mapping quality, library complexity, ChIP enrichment, fraction of reads in peaks, and overlap with DNase hypersensitive sites), and this dataset passed all QC metrics; therefore, we used the BED file available from the CistromeDB website. To increase confidence in p53 ChiP-seq peaks that correlate with regions of productive transcription, we used bedtools intersect with the flag “−F 0.5” to only retain ChIP peaks that overlapped at least 50% with regions of productive transcription. This reduced the number of p53 ChIP peaks from 13,453 to 2,163. We then used the featureCounts module in R to count the number of ATAC-seq reads mapped to these peaks and performed differential accessibility analysis using DESeq2. The list of significant (*P*_adj_ ≤ 0.1) hits was then manually curated to select for regions that were proximal to or within genes undergoing bidirectional transcription using the Nutlin-3A PRO-seq dataset. Of these, we selected six candidates whose target genes were of interest and lent themselves to successful qPCR primer validation via amplification efficiency testing.

The six candidates were selected based on the following criteria: (1) they exhibited differential accessibility in at least one of the Zn^2+^ perturbations, (2) they overlapped a p53 binding site identified by ChIP-seq, (3) they correlated with regions of active transcription upon activation of p53 by nutlin 3a, (4) primers could be designed within the introns of the gene or at most ∼1,000 base pairs up/downstream of the gene. Within the p53 targets in this list is an intronic region of endoplasmic reticulum-Golgi intermediate compartment 1 (*ERGIC1*), which encodes a cycling membrane protein whose potential function is to transport cargo from the ER to the Golgi (Breuza et al, 2004). In TPA treated cells, the accessibility at this genomic region was slightly, but significantly increased, whereas treatment with ZnCl_2_ did not significantly alter its accessibility. In addition, an intronic region of the Nuclear Factor I B (*NFIB*), a TF that regulates differentiation and proliferation of the central nervous system (Chen et al, 2017), showed significantly reduced accessibility with TPA. We also targeted a p53 binding site upstream of stratifin (*SFN*), which, when activated by p53 because of detected DNA damage, leads to cell-cycle arrest at the G2/M transition (Taylor & Stark, 2001; Arakawa et al, 2022). This region saw a significant increase in accessibility in TPA. A region that saw a large increase in accessibility under ZnCl_2_ conditions was a p53 binding site downstream of the early growth response 1 (*EGR1*) gene, which encodes a TF containing three C2H2-type zinc fingers that can regulate p53 activity upon ionizing radiation exposure (Inoue & Fry, 2018). Cells treated with ZnCl_2_ also displayed a slight but significant reduction in the accessibility at the intronic regions of *PLD5* and *LRIG3-DT*. Phospholipase D family member 5 (*PLD5*) has been shown to be regulated by a tumor suppressor microRNA that is itself a client of p53 (Liu et al, 2021), whereas the leucine-rich repeats and immunoglobulin-like domains divergent transcript (*LRIG3-DT*) encodes an integral plasma membrane protein whose expression has been shown to positively correlate with the expression of p53 in cervical intraepithelial neoplasia (Lindström & Hellberg, 2014).

As a positive control for immunoprecipitation, we used previously published (Menendez et al, 2013) primers targeting the p53 response element within the promoter of *CDKN1A* (p21), a well-established p53 target (Macleod et al, 1995; He et al, 2005). For *CDKN1A*, the p53 binding site was not included in our DESeq2 analysis, as the primary region of active transcription within the p53 PRO-seq dataset was downstream of the p53 binding site we chose. Visually, however, the region appeared to be slightly more accessible in TPA. As a negative control for immunoprecipitation, the Human Negative Control Primer Set 1 (#71001; ActiveMotif) amplifies a 78 base pair fragment from a gene desert on human chromosome 12 and would not be expected to show enrichment when incubated with the p53 antibody. This primer set is recommended as a negative control for almost all transcription factors.

### ChIP-qPCR

Wildtype MCF10A cells (2 × 15 cm dishes with approximately 12 million cells/dish) were treated as noted above for ATAC-seq for 30 min, followed by washing with room temperature PBS, crosslinking using 1% methanol-free formaldehyde (#28908; Thermo Fisher Scientific) for 10 min and quenching for 8 min using 125 mM glycine. Cells were washed using cold PBS and nuclei were isolated using a hypotonic buffer (10 mM Tris–HCl pH 7.5, 50 mM NaCl, 2 mM EDTA, 1% NP-40, 1 mM DTT, and 1x protease inhibitors [#78441; Thermo Fisher Scientific]). Nuclei were then lysed and subjected to chromatin fragmentation using a BioRuptor (Diagenode) sonicator (7 x 10 min pulses, high setting) resulting in a mean chromatin length of ∼200 bp, followed by a spin at 21,130*g* for 15 min at 4°C to pellet insoluble material. Chromatin was aliquoted, flash frozen in liquid nitrogen, and stored at −70°C.

To immunoprecipitate p53-bound chromatin, 220 μl of chromatin (from approximately 7 × 10^6^ MCF10A cells) were pre-cleared per condition by incubating with 30 μl of Protein A/G beads (#SC-2003; Santa Cruz. #B2323; Lot) for 2 h at 4°C on a rotator. Then, 1/10^th^ of the pre-cleared chromatin from each condition was saved as “Input” and flash frozen, and half of the remaining chromatin from each condition was rotated overnight at 4°C with 4.5 μg of anti-p53 antibody (#554293; BD Biosciences, #9046678; Lot). The other half of the remaining chromatin from each condition was also rotated overnight with no antibody. Next, chromatin samples (+/− antibody for TPA, Control, and ZnCl_2_) were incubated for 2 h with Protein A/G beads (15 μl of beads per 100 μl of chromatin; blocked overnight with 0.5 mg/ml bovine serum albumin and 0.4 mg/ml yeast RNA). The beads were then washed (resuspension followed by centrifugation) twice with 400 μl of each a low salt buffer (20 mM Tris–HCl pH 8, 150 mM NaCl, 2 mM EDTA, 1% Triton X 100, 0.1% SDS), a high salt buffer (20 mM Tris–HCl pH 8, 500 mM NaCl, 2 mM EDTA, 1% Triton X-100, 0.1% SDS), a high salt buffer containing LiCl (20 mM Tris–HCl pH 8, 250 mM LiCl, 1. mM EDTA, 1% sodium deoxycholate, 1% NP-40), and finally with TE buffer (10 mM Tris–HCl pH 8, 1 mM EDTA). Beads were then resuspended in 200 μl of elution buffer (50 mM Tris–HCl pH 8, 10 mM EDTA, 1% SDS) and incubated for 1 h at 37°C with shaking. Frozen inputs were thawed and diluted to a total volume of 200 μl of elution buffer and incubated for 1 h at 37°C with shaking. To reverse crosslinks, the resulting supernatant from chromatin samples and inputs were incubated overnight in 200 mM NaCl at 65°C. Next, samples were diluted 1:1 with Milli Q water to lower the salt concentration and proteins were digested using 160 μg of Proteinase K at 55°C for 1 h. DNA was purified via phenol:chloroform:isoamyl alcohol (#15593031; Thermo Fisher Scientific) extraction and ethanol precipitation. DNA pellets of chromatin samples (+/− antibody for TPA, Control, and ZnCl_2_) and of inputs were then resuspended in 27 or 100 μl of diethylpyrocarbonate (DEPC)-treated water, respectively.

For qPCR, 10-fold serial dilutions (1:10, 1:100, 1:1,000) of each input from each condition were prepared to allow for an absolute quantification analysis of the data. The qPCR reactions were prepared as follows: 600 nM forward primer, 600 nM reverse primer, 3 μl of DNA (sample, diluted input, or a no-treatment control [DEPC-water]), and 1X SYBR Select qPCR master mix (#4472918; Thermo Fisher Scientific), with a final volume of 21 μl. qPCR was then performed in technical duplicate (10 μl/reaction) on a Bio-Rad C1000 Touch thermal cycler using the recommended SYBR Select protocol and an annealing temperature of 57°C. C_T_ values between technical replicates were averaged and the %IP for each chromatin sample and primer set was calculated by generating a standard curve of the log quantities (based on dilution factor) of input chromatin versus their C_T_ values, performing a regression analysis to calculate the quantity of DNA in the sample, and finally accounting for all dilutions made throughout the ChIP protocol given the equation: $\%IP=\left(DNA\;quantity\right)*\frac{\left(Volume\;of\;ChIP\;eluate\right)}{\left(Volume\;of\;input\;eluate\right)}*\frac{\left(Volume\;of\;chromatin\;used\;for\;ChIP\right)}{\left(Volume\;of\;chromatin\;used\;as\;input\right)}$. All qPCR primers used in this study are listed in Table S3. The entirety of the ChIP-qPCR protocol was run on six biological replicates.

Table S3. List of ChIP-qPCR primers used in this study.

The signal:noise was calculated by dividing the %IP in the +Ab sample by the %IP in the −Ab sample for each target. The enrichment ratio was calculated by dividing the %IP in the +Ab sample for a given target by the %IP in the +Ab sample for the respective control sample. An inter-quartile statistical test was run on the enrichment ratio data to determine statistical outliers.

## Supplementary Material

Reviewer comments

## Data Availability

All raw and processed sequencing data generated in this study have been submitted to the NCBI Gene Expression Omnibus (GEO; https://www.ncbi.nlm.nih.gov/geo/) under accession number GSE212763.
