# Supplementary material for: Cellular zinc status alters chromatin accessibility and binding of p53 to DNA
Source: Life Sci Alliance. 2024 Jul 8;7(9):e202402638. doi: 10.26508/lsa.202402638 (PMC11231577; doi:10.26508/lsa.202402638)
Supplement: Supplementary file 7 [file LSA-2024-02638_TableS3.docx]

| Target | Orientation | Primer Sequence |
| --- | --- | --- |
| CDKN1A (p21 5’) | Fwd | AGC AGG CTG TGG CTC TGA TT |
| CDKN1A (p21 5’) | Rev | CAA AAT AGC CAC CAG CCT CTT CT |
| EGR1 | Fwd | GCC TGA GGT TCT AGG TTC TTT |
| EGR1 | Rev | TCA AGG CCT CTC ACA GAG TA |
| ERGIC1 | Fwd | CAG TTG AAT GAG TGT CCT TTG G |
| ERGIC1 | Rev | ATG GCT AGC GAC TTC AGT G |
| Human Negative Control Set 1 | Fwd + Rev | Proprietary (ActiveMotif, #71001) |
| LRIG3-DT | Fwd | TTG GAA AGC ACA CGC GAT AA |
| LRIG3-DT | Rev | CTG ACT TGT TAG AGC ATG TCA CAG |
| NFIB | Fwd | CTC TTG CTC CCT CCA CCT |
| NFIB | Rev | GGA TTG GGA GCC AGC CA |
| PLD5 | Fwd | TTC AAC CTG TGG TGA GTC TAA G |
| PLD5 | Rev | CCA AGA CGT CTC AAA GCT ACT |
| SFN | Fwd | TTC CCA GCC TCA GAC AGA |
| SFN | Rev | GCC ACA TGC CAT CAG GTA |
